# Anesthetic Considerations for Removal of Bilateral Orbital Foreign Bodies With Suspected Carotid Artery Involvement: A Case Report

**DOI:** 10.7759/cureus.102094

**Published:** 2026-01-22

**Authors:** Asad Saleem, Illan Kunik, Jay D Shah, Noureddeen Bitar, Anvinh Nguyen

**Affiliations:** 1 Department of Anesthesiology, Baylor College of Medicine, Houston, USA

**Keywords:** anesthesiology, carotid artery injury, intraocular pressure, open globe injury, penetrating ocular trauma, rapid sequence induction, video laryngoscopy

## Abstract

Penetrating ocular trauma and carotid artery involvement each present distinct anesthetic challenges. In patients with suspected open globe injuries, increases in intraocular pressure must be minimized through careful anesthetic and airway management. Concurrent carotid artery involvement carries the risk of catastrophic hemorrhage and requires invasive monitoring and multidisciplinary coordination.

We report the anesthetic management of a 74-year-old male presenting with bilateral orbital penetration by ballpoint pens, with imaging revealing the right pen abutting the internal carotid artery. This case highlights key anesthetic strategies and the importance of coordinated endovascular and surgical planning to ensure patient safety.

## Introduction

Penetrating ocular trauma is a vision-threatening emergency that may result in open globe injury and frequently necessitates urgent surgical intervention to prevent permanent visual impairment or blindness [1]. Ocular trauma remains a significant cause of monocular blindness worldwide, with penetrating mechanisms associated with worse visual outcomes compared with blunt injury [2]. Penetrating injuries to the orbit account for up to 50% of traumatic orbital injuries and are particularly concerning due to the dense concentration of critical ocular, vascular, and intracranial structures within a confined anatomic space [3].

The anesthetic management of penetrating orbital trauma is uniquely challenging. Anesthesiologists must balance the need for rapid airway control in patients with uncertain fasting status against the imperative to avoid increases in intraocular pressure (IOP), which may exacerbate extrusion of ocular contents and worsen visual prognosis in open globe injuries [4]. Perioperative factors such as coughing, bucking, vomiting, hypertension, and patient movement can all acutely increase IOP and compromise surgical repair [4]. In addition, airway manipulation, mask ventilation, and patient positioning may inadvertently result in migration of penetrating foreign bodies, potentially converting a stable injury into a catastrophic one.

These challenges are amplified when penetrating orbital trauma involves proximity to major vascular or intracranial structures. Orbital foreign bodies have been reported to traverse the superior orbital fissure or optic canal, placing the internal carotid artery (ICA), cavernous sinus, and brain at risk for hemorrhage, stroke, or death [5,6]. In such cases, anesthetic missteps during induction, maintenance, or emergence may precipitate devastating complications, underscoring the importance of meticulous planning, hemodynamic control, and close coordination among anesthesia, ophthalmology, neurointerventional radiology, neurosurgery, and trauma teams.

We report the case of a 74-year-old male who sustained bilateral penetrating orbital trauma from ballpoint pens, with computed tomography demonstrating that one foreign body was abutting the right ICA. This rare and high-risk presentation highlights critical anesthetic considerations in emergent penetrating orbital trauma, particularly the need to prevent foreign body displacement, maintain stable intraocular and cerebral perfusion pressures, and ensure a smooth induction and emergence. Through this case, we aim to emphasize key anesthetic principles that may mitigate the risk of catastrophic hemorrhage while optimizing conditions for surgical repair and patient survival.

## Case presentation

**Figure 1:**
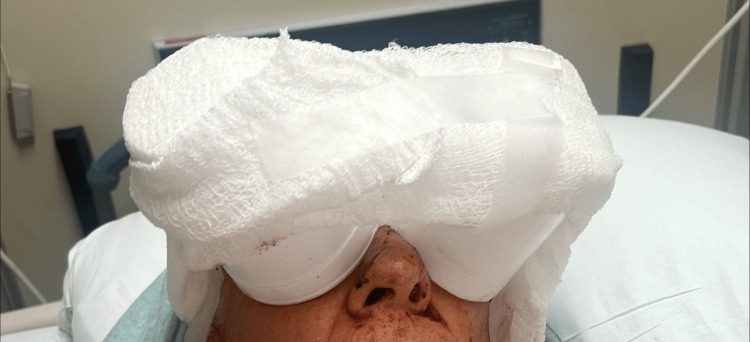
Protective eye shields placed over both orbits on presentation to prevent inadvertent manipulation or advancement of penetrating foreign bodies prior to surgical intervention.

A 74-year-old male presented to our community hospital with bilateral traumatic pens penetrating each orbit. The patient weighed 90 kg and was 177 cm tall. Other relevant past medical history included a cerebrovascular accident in 2024 with residual visual deficits in the right eye, hypertension, bipolar II disorder, and severe major depressive disorder with active suicidal ideation. He took lisinopril at home and received levofloxacin and vancomycin preoperatively. Preoperative physical examination showed two cups over the bilateral eyes due to concern for open globe injury and accidental advancement of the pens (Figure 1).

The preoperative exam was remarkable for Mallampati III, limited neck range of motion due to discomfort, blood-tinged teeth, and an alert and awake patient. Preoperative labs were unremarkable except for a mildly elevated glucose of 146 mg/dL (reference range 70-110 mg/dL).

**Figure 2:**
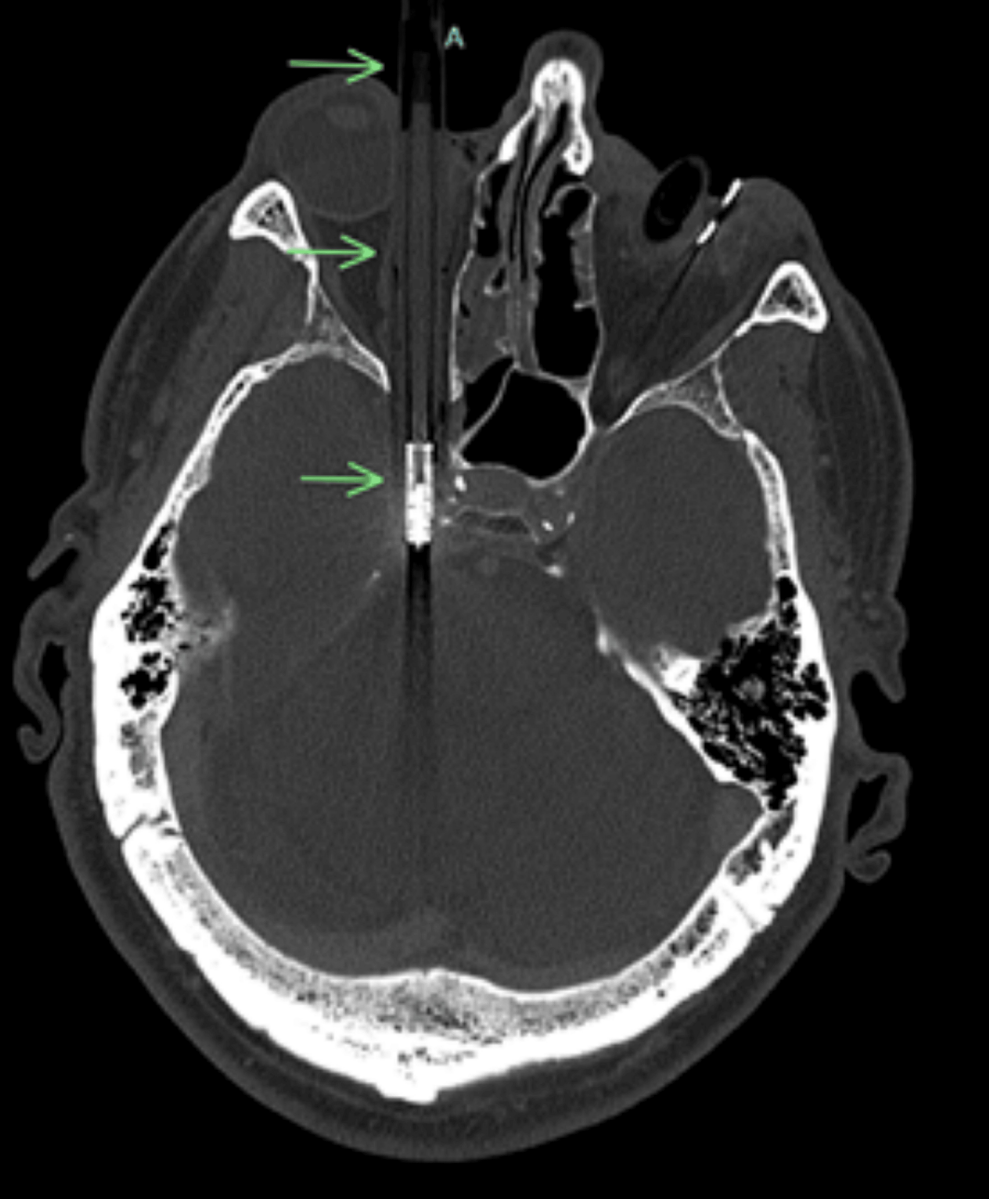
Axial computed tomography image demonstrating a ballpoint pen penetrating the right orbit, extending posteriorly toward the cavernous sinus region.

**Figure 3:**
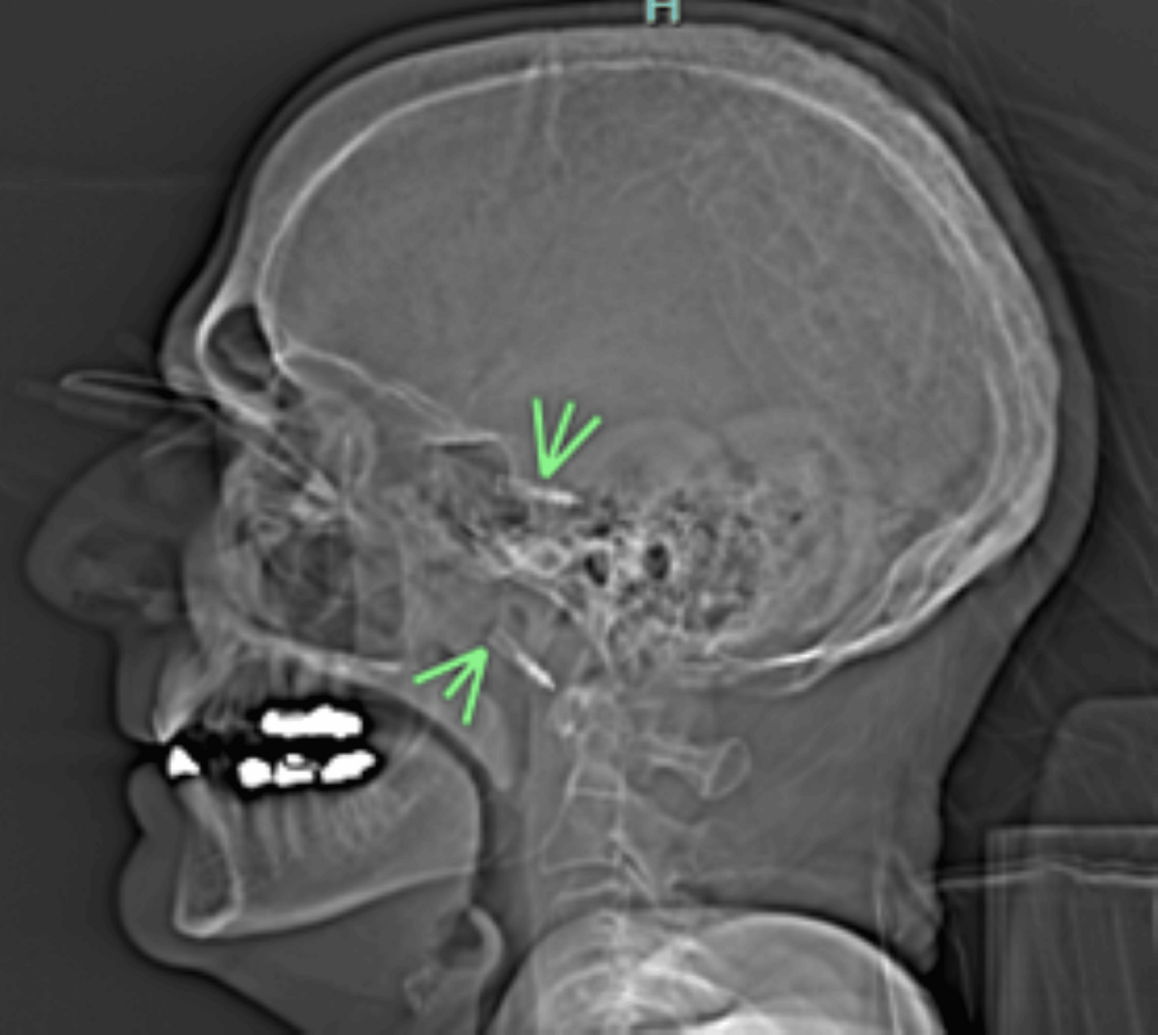
Sagittal computed tomography image showing bilateral orbital penetration by ballpoint pens with intracranial extension.

Computed tomography with angiography (CTA) of the head demonstrated near-complete occlusion of the right cavernous ICA (Figure 2 and Figure 3). A trace hematoma was seen in the right eye around the right pen. Bilateral globes were grossly intact on CT scan. On imaging, it was not possible to assess for intimal injury to the right ICA per the radiology report. An electrocardiogram or transthoracic echocardiogram was not available.

Prior to proceeding to the operating room, a multidisciplinary preoperative discussion was held to determine the optimal surgical sequence. The plan involved initial endovascular intervention by the neurointerventional radiology team to place protective coils in the arteries adjacent to the right ICA, followed by removal of the orbital foreign bodies by the ophthalmology team. Neurosurgery and otolaryngology were placed on standby to assist in the event of intraoperative vascular or structural complications.

**Figure 4:**
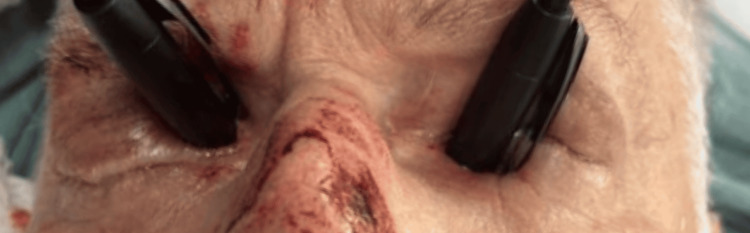
Intraoperative photograph demonstrating bilateral orbital penetration by foreign bodies immediately prior to anesthetic induction and airway management.

No premedication was administered prior to transport to the operating room. On arrival, the patient was noted to be mildly hypertensive with a blood pressure of 171/100 mmHg, heart rate of 79 beats per minute, respiratory rate of 19 breaths per minute, and oxygen saturation of 99% on room air. The protective cups over both eyes were carefully removed to facilitate airway management and tracheal intubation (Figure 4).

Preoxygenation was performed with 100% oxygen via a face mask for five minutes of tidal breathing to replace alveolar nitrogen and increase oxygen reserves, thereby prolonging safe apnea time during induction. A rapid sequence induction (RSI) was performed to minimize the risk of aspiration given the patient’s inadequate NPO status. Additionally, RSI was selected to avoid positive-pressure ventilation, as bag-mask ventilation could potentially displace or advance the orbital foreign bodies. Induction was achieved with intravenous fentanyl 150 µg, lidocaine 100 mg, propofol 170 mg, and rocuronium 110 mg. Video laryngoscopy (VL) with a McGrath size 3 blade provided a clear view of the vocal cords. Extreme caution was exercised to prevent head and neck movement during airway manipulation due to the proximity of the penetrating objects. An 8.0 mm endotracheal tube was advanced through the glottic opening, and correct placement was confirmed by continuous waveform capnography.

Following induction, a right radial arterial line was inserted under ultrasound guidance without complication. In anticipation of a potential massive hemorrhage during the removal of the orbital foreign bodies, a Belmont rapid infuser was prepared, and two units of cross-matched packed red blood cells were made immediately available in the operating room.

**Figure 5:**
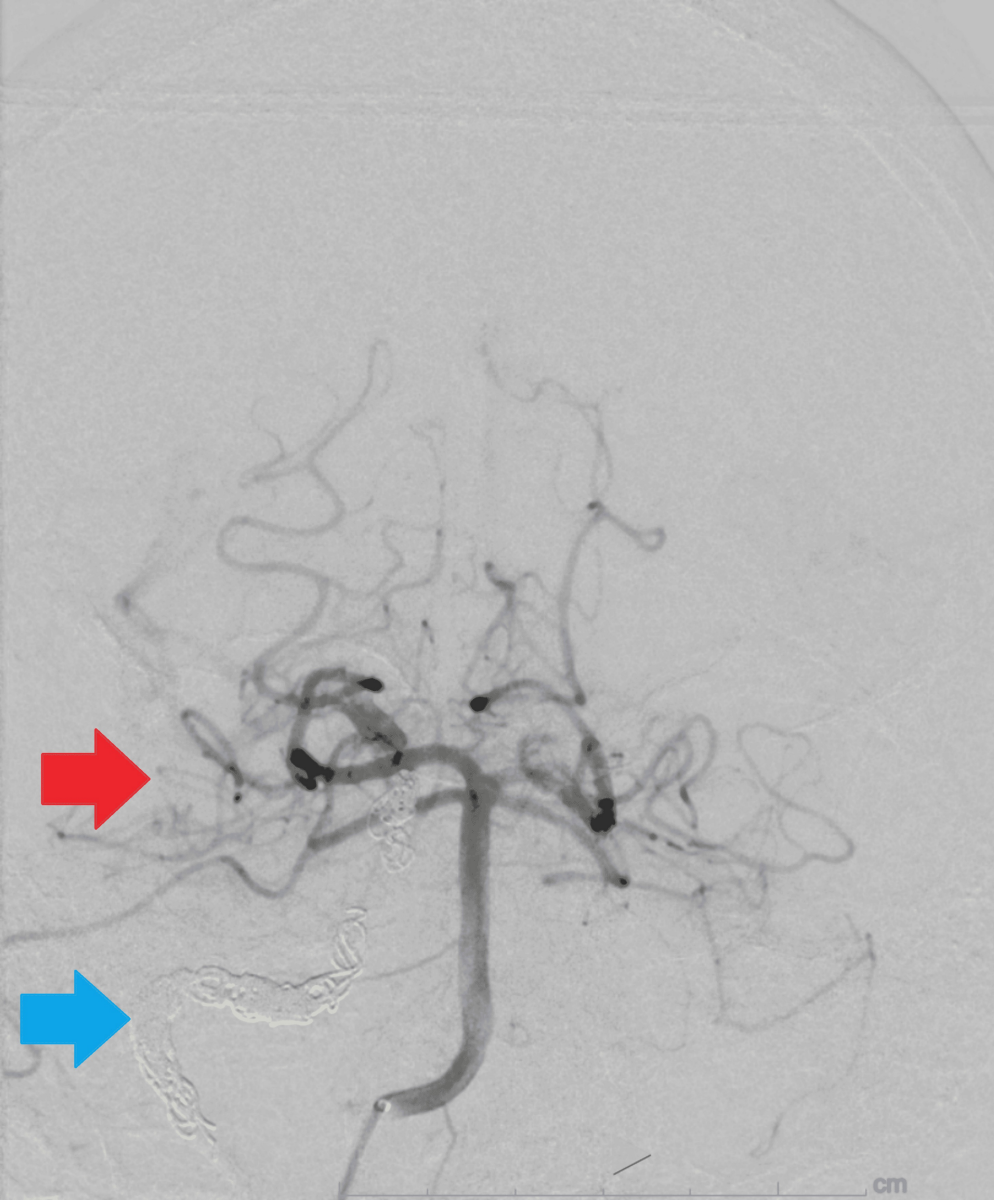
Repeat angiography confirmed occlusion of the right internal carotid artery (blue arrow) and adequate collateral perfusion to the right middle cerebral artery (red arrow).

The neurointerventional radiology team initiated the procedure by catheterizing the right femoral artery and accessing the right common carotid artery. Two Penumbra packing coils were deployed into the petrous segment of the vessel. Repeat angiography confirmed occlusion of the right ICA (blue arrow) and demonstrated adequate collateral perfusion to the right middle cerebral artery (red arrow) via the anterior cerebral and bilateral ophthalmic arteries (Figure 5).

Ophthalmology then proceeded with the removal of the foreign bodies. The pen in the left orbit was removed first without significant blood loss. The second pen was subsequently extracted from the right orbit, resulting in brisk bleeding that was controlled with fibrin-soaked sponges. The ophthalmologic examination confirmed no evidence of open globe injury, and both orbital wounds were repaired without complication.

Anesthesia was maintained with sevoflurane at approximately 1.0 minimum alveolar concentration (MAC), and additional rocuronium was administered to ensure complete neuromuscular paralysis and minimize IOP fluctuations during ocular manipulation. At the conclusion of the procedure, neuromuscular blockade was reversed with sugammadex 180 mg, and the patient was extubated under a deep plane of anesthesia to facilitate a smooth, controlled emergence and reduce the risk of coughing or bucking.

The patient’s postoperative course was otherwise unremarkable, with preservation of bilateral visual function. He was discharged in good condition on postoperative day five, with outpatient follow-up with ophthalmology arranged prior to discharge.

## Discussion

Given the complexity of the patient’s injuries, careful planning and coordination among multiple surgical specialties were essential. In addition to ophthalmology, the multidisciplinary team included neurointerventional radiology, neurosurgery, and otolaryngology. Because the patient was hemodynamically stable and not acutely decompensating, there was an opportunity for preoperative planning to delineate the sequence of critical interventions. Due to the proximity of one of the foreign bodies to the right ICA, the team determined that neurointerventional radiology would perform endovascular coiling prior to ophthalmologic extraction. This approach was designed to mitigate the risk of catastrophic hemorrhage in the event of carotid injury during removal. Neurosurgery remained on standby to provide immediate intervention in the event of intracranial bleeding, and otolaryngology was available due to imaging findings suggesting possible extension into the nasal cavity.

Intraoperative management of patients with suspected open globe injuries focuses on minimizing increases in IOP and preventing aspiration, particularly in the setting of traumatic injury. Elevations in IOP are most pronounced during induction, intubation, and emergence [7]. Regional anesthetic techniques, such as retrobulbar or peribulbar blocks, are often avoided because they can exacerbate IOP. Agents such as succinylcholine and ketamine may also increase IOP and are typically avoided, whereas propofol, opioids, and volatile anesthetics have a beneficial IOP-lowering effect [4]. In this case, we performed an RSI with propofol, fentanyl, and rocuronium, followed by maintenance with sevoflurane, to optimize hemodynamic stability while minimizing IOP fluctuations.

Additionally, care must be taken when ventilating the patient with a face mask to avoid pressure on the eye. In this case, as the patient had pens protruding from bilateral eyes, further care was required to ensure no contact was made with either pen during intubation to prevent movement of the pens within the patient’s orbits, further propagating injury. A rapid sequence intubation was performed so the patient did not have a face mask applied during induction.

During laryngoscopy, IOP doubles on the first attempt and increases by an additional 30% on the second attempt [4]. A study comparing the Macintosh blade to VL showed a more significant increase in IOP with the Macintosh blade compared to the VL group [4]. In our case, we chose to intubate under VL to both minimize manipulation of the head and neck with pens protruding from bilateral eyes and to decrease the chance of increasing IOP during intubation.

Coughing and bucking during extubation can significantly increase IOP and hemodynamic stress. Various strategies, including administration of sympatholytics such as opioids, intravenous lidocaine, or dexmedetomidine, as well as deep extubation, can help mitigate these responses [4]. In our case, although ophthalmologic evaluation revealed no open globe injury following removal of the pens, we implemented these precautions to ensure a smooth and controlled emergence.

Careful assessment and management of potential carotid artery injury are critical due to the risk of abrupt intraoperative hemodynamic compromise. For this patient, the initial CTA could not fully delineate the extent of injury to the right ICA. It remained uncertain whether the pen had caused a true vascular injury with risk of bleeding upon removal, or if it was merely abutting or tenting the vessel without disruption of the intimal layer. This uncertainty informed our decision for preemptive neurointerventional coiling and vigilant intraoperative monitoring.

When assessing vascular injuries, especially neck injuries with concern for the carotid artery, emergent neck exploration is indicated if there is evidence of “hard signs” of vascular injury. “Hard signs” of vascular injury can include evidence of pulsatile or expanding neck hematoma, external hemorrhage, tracheal deviation, or acute neurological deficits. Without evidence of hard signs of vascular injury, time and preparation can be taken for further assessment of the injury [8]. Given the uncertainty of the vascular injury, we ensured large-bore IV access prior to surgical intervention (meaning at least two 18-G IVs) and invasive blood pressure monitoring with a post-induction arterial line placement for close hemodynamic monitoring. A rapid infuser was also readily available in the room if rapid transfusion became necessary.

## Conclusions

Open globe injuries and carotid artery involvement each present distinct and significant anesthetic challenges. Preventing intraoperative elevations in IOP requires deliberate planning to ensure smooth induction, atraumatic airway management, adequate depth of anesthesia, and controlled emergence, as even transient increases in pressure may compromise globe integrity and visual outcomes. Simultaneously, the possibility of carotid artery injury mandates vigilant hemodynamic assessment, invasive monitoring when appropriate, and readiness for rapid transfusion and resuscitation in the event of catastrophic hemorrhage.

When penetrating orbital trauma places multiple critical structures at risk, anesthetic management extends beyond routine considerations and demands a highly coordinated, multidisciplinary approach. Close collaboration among anesthesiology, ophthalmology, neurosurgery, vascular surgery, trauma surgery, and radiology is essential to minimize the risk of foreign body migration, optimize surgical conditions, and respond swiftly to life-threatening complications. This case illustrates how meticulous preoperative planning, clear intraoperative communication, and thoughtful anesthetic execution can contribute to successful foreign body removal while preserving both vision and life. It underscores the central role of the anesthesiologist in managing complex orbital trauma and highlights key principles that may guide safe perioperative care in similarly high-risk scenarios.
